# Satellite-Based and Street-View Green Space and Adiposity in US Children

**DOI:** 10.1001/jamanetworkopen.2024.49113

**Published:** 2024-12-05

**Authors:** Li Yi, Soren Harnois-Leblanc, Sheryl L. Rifas-Shiman, Esra Suel, Marcia Pescador Jimenez, Pi-I Debby Lin, Perry Hystad, Steve Hankey, Wenwen Zhang, Marie-France Hivert, Emily Oken, Izzuddin M. Aris, Peter James

**Affiliations:** 1 Department of Population Medicine, Harvard Medical School and Harvard Pilgrim Health Care Institute, Boston, Massachusetts; 2 Centre for Advanced Spatial Analysis, University College London, London, United Kingdom; 3 Department of Epidemiology, Boston University School of Public of Health, Boston, Massachusetts; 4 College of Public Health and Human Sciences, Oregon State University, Corvallis; 5 School of Public and International Affairs, Virginia Tech, Blacksburg; 6 Edward J. Bloustein School of Planning and Public Policy, Rutgers, The State University of New Jersey, New Brunswick; 7 Diabetes Unit, Massachusetts General Hospital, Boston; 8 Department of Nutrition, Harvard T.H. Chan School of Public Health, Boston, Massachusetts; 9 Department of Public Health Sciences, University of California Davis School of Medicine, Davis; 10 Department of Environmental Health, Harvard T.H. Chan School of Public Health, Boston, Massachusetts

## Abstract

**Importance:**

Green space may be associated with obesity outcomes in children by promoting physical activity and reducing stress. However, previous studies have mostly used a single measure of green space and body mass index (BMI) as the sole indicator of adiposity.

**Objective:**

To examine associations of the satellite-based normalized difference vegetation index (NDVI) and street-view imagery–based green space metrics with comprehensive adiposity measures in children.

**Design, Setting, and Participants:**

The Project Viva cohort study observed participants from mid-childhood (mean [SD] age, 8.0 [0.8] years) and early adolescence (mean [SD] age, 13.3 [1.0] years) through late adolescence (mean [SD] age, 17.8 [0.7] years) between January 1, 2007, and December 31, 2021. This analysis included children with data on green space and adiposity measures. Data were analyzed from January 1 to June 30, 2024.

**Exposures:**

Green space metrics (ie, percentage of trees, grass, and other green space) using street-view imagery and deep learning algorithms along with the NDVI were linked with participants’ home addresses during mid-childhood and early adolescence.

**Main Outcomes and Measures:**

Body mass index (calculated as weight in kilograms divided by height in meters squared) and waist circumference *z* scores were calculated from anthropometric measurements during early and late adolescence. The total fat mass index (FMI; calculated as fat mass in kilograms divided by height in meters squared) and trunk FMI were obtained using dual-energy x-ray absorptiometry. Separate linear regression models adjusted for a child’s age, sex, individual and neighborhood socioeconomic status, and population density estimated prospective associations of the NDVI and street-view green space (in mid-childhood or early adolescence) with 4 adiposity measures (in early or late adolescence).

**Results:**

The sample included 843 children (mean [SD] age at mid-childhood, 7.9 [0.8] years; 423 girls [50.2%]) at baseline. After adjustment for confounders, a higher NDVI (per 1-SD increase) within 270 m in early adolescence was associated with lower *z* scores for BMI (−0.16 [95% CI, −0.27 to −0.04]) and waist circumference (−0.13 [95% CI, −0.23 to −0.03]), as well as a lower total FMI (−0.62 [95% CI, −1.02 to −0.23]) and trunk FMI (−0.33 [95% CI, −0.55 to −0.12]) in late adolescence. Higher percentages of street-view other green space (flowers, plants, and fields), but not street-view trees and grass, within 500 m in early adolescence were associated with lower BMI *z* scores (−0.09 [95% CI, −0.18 to −0.01) in late adolescence. The NDVI and street-view green space exposure at mid-childhood were not associated with adiposity measures in early adolescence.

**Conclusions and Relevance:**

This cohort study of US children suggests that higher levels of satellite-based NDVI greenness and percentages of street-level green space components (flowers, plants, and fields) were associated with lower adiposity. The findings support the exploration of increasing residential green space levels and adding specific green space components as an urban planning and public health intervention strategy to combat the prevalence of childhood obesity in the US.

## Key Points

**Question:**

Is residential green space exposure, assessed using the satellite-based normalized difference vegetation index (NDVI) and street-view imagery, associated with adiposity measures from childhood to adolescence?

**Findings:**

In this US-based cohort study of 843 children, a higher satellite-based NDVI in early adolescence was associated with lower body mass index (BMI), waist circumference, and total body and trunk fat mass in late adolescence. More street-view other green space components (flowers, plants, and fields) were also associated with a lower BMI in the same period.

**Meaning:**

This study suggests that increasing the overall residential green space and specific green space components could potentially mitigate the prevalence of childhood obesity.

## Teaser

This cohort study examines associations of residential green space exposure, assessed using the satellite-based normalized difference vegetation index and street-view imagery, with comprehensive adiposity measures from childhood to adolescence.

## Introduction

Pediatric obesity is associated with numerous physical and psychosocial consequences as well as decreased quality of life.^1^ Recent studies suggest that access to residential green space is associated with a lower risk of obesity in pediatric populations by reducing exposure to air pollution and noise; promoting physical activity, social engagement, and sleep; and directly reducing stress.^2,3,4,5,6,7^ However, findings on residential green space and obesity are inconsistent.^8^

Results may be inconsistent because previous studies have predominantly used satellite-based measures of greenness, such as the normalized difference vegetation index (NDVI).^8,9^ However, the NDVI does not differentiate between types of green space, such as trees, grass, or other types of vegetation, which may have different associations with obesity and thus may incorporate exposure misclassification as well as different confounding structures into study results.^10^ Recently, street-view–based measures have emerged to complement the NDVI to examine associations between individual green space components and obesity.^5,11,12,13^ However, few studies^14,15^ have examined the associations of both the satellite-based NDVI and street-view green space components with obesity, and none of these studies have focused on children.

Moreover, previous studies on the association between the NDVI and obesity in children have relied primarily on body mass index (BMI; calculated as weight in kilograms divided by height in meters squared),^8^ which has limited ability to accurately reflect body fat accumulation.^16^ More accurate adiposity measures, such as total fat mass and trunk fat derived from dual-energy x-ray absorptiometry and waist circumference, would better ensure long-term cardiometabolic health.^17^ Few studies^18,19,20^ have examined the association between residential green space and these adiposity measures. Furthermore, most studies are cross-sectional and focused on the adult population, limiting our understanding of the temporal associations of green space exposure with childhood obesity.^21,22^

To address these gaps, we applied deep learning segmentation algorithms to quantify specific components of green space from street-view imagery and linked these data along with the satellite-based NDVI to the residential addresses of children in the Project Viva study. We then examined the prospective association of the NDVI and street-view metrics with a comprehensive set of adiposity measures from mid-childhood to late adolescence (2007-2021). We hypothesized that street-level green space metrics would better capture green space exposures important to childhood obesity and would therefore have a stronger inverse association with adiposity than the satellite-based NDVI.

## Methods

### Study Population

We used data from Project Viva, a prebirth, US-based cohort. Between 1999 and 2002, Project Viva recruited pregnant women from a large group practice in eastern Massachusetts and has followed up with mother-child pairs since pregnancy, even if they relocated outside of Massachusetts. Initially, we recruited 2128 liveborn singleton infants; among them, 1279 (60.1%) participated in the mid-childhood (mean [SD] age, 8.0 [0.8] years) in-person visit (2007-2011), 1177 (55.3%) participated in the early adolescence (mean [SD] age, 13.3 [1.0] years) visit (2012-2016), and 809 (38.0%) participated in the late adolescence (mean [SD] age, 17.8 [0.7] years) visit (2017-2021). Our final analysis sample included 843 children (39.6%) who had both green space exposure metrics in mid-childhood and adiposity measures in early adolescence and 599 children (28.1%) who had both green space exposure metrics in early adolescence and adiposity measures in late adolescence (see flowchart in eFigure 1 in Supplement 1). All mothers provided written informed consent at each visit, and children provided verbal informed consent from mid-childhood; written informed consent was obtained when they reached 18 years of age. The institutional review board of Harvard Pilgrim Health Care approved the study protocols. This cohort study followed the Strengthening the Reporting of Observational Studies in Epidemiology (STROBE) reporting guideline.

### Green Space Exposure

#### Street-View Green Space Metrics

We applied pyramid scene parsing network (PSPNet, extension number CVPR2017)^23^ deep learning algorithms to street-view images every 100 m along the street network for all cities in the US from 2007 to 2020 (the methods are described in detail in previous studies^5,24^ and briefly in the eMethods, eFigure 9, and eTable 4 in Supplement 1). We then linked 100-m metrics with geocoded participant addresses across the US obtained in mid-childhood and early adolescence to derive street-view green space exposure metrics for the corresponding year (eg, linking an address from 2007 with street-view data from 2007). We also derived street-view metrics based on all images within the 500-m and 1000-m buffers of each address. The 3 green space exposure metrics included the percentage of trees (trees and palm trees), grass, and other green space (flowers, plants, and fields). We internally standardized all metrics to *z* scores, which can be interpreted as per SD increments, and allowed for direct comparison between different metrics. We used 500 m as the primary exposure to represent typical spatial extents of children’s residential neighborhoods and 100 m and 1000 m as sensitivity analyses.^25^

#### Satellite-Based NDVI

We also linked 30-m Landsat NDVI estimates (range, 0-1 for July of the respective year in which the visit took place) with participants’ addresses at the mid-childhood and early adolescence visits. We then derived NDVI estimates for the 90-m, 270-m, and 1230-m buffers. The NDVI was also internally standardized to *z* scores to allow for comparison with street-view metrics. We used the 270-m NDVI as the primary exposure buffer size that was closest to the 500-m street-view metrics, and we used 90 m and 1230 m as sensitivity analyses that were closest to the 100-m and 1000-m street-view metrics, respectively.

### Adiposity Outcomes

We investigated the association between green space exposure and 4 adiposity measures: BMI, waist circumference, total body fat mass, and trunk fat mass. We chose these measures to verify the consistency in the association across all indicators, thereby reinforcing our conclusions. In addition, we aimed to assess whether green spaces were differentially associated with total body fat vs abdominal fat and whether these associations could be identified using clinically common measures.^17,26^ At the early adolescence and late adolescence visits, trained research staff measured the child’s weight with a Tanita scale and height with a stadiometer and calculated BMI sex- and age-specific *z* scores.^27^ Project Viva staff measured the child’s waist circumference at the midaxillary line above the iliac crest to the nearest 0.1 cm.^27^ Because waist circumference in children is highly dependent on sex and developmental stage, we calculated age- and sex-specific waist circumference *z* scores.^28^ We assessed total body and trunk fat mass in a subsample of children using dual-energy x-ray absorptiometry (Discovery A; Hologic Inc), and we assessed total body and trunk fat mass indices (FMIs; calculated as fat mass in kilograms divided by height in meters squared).

### Covariates

We obtained data on the sex of the child from the delivery hospital medical records. During the late adolescence visit, children reported their race and ethnicity (if missing, the race and ethnicity reported by the mother was used), which we categorized as Hispanic, non-Hispanic Black, non-Hispanic White, or other (American Indian or Alaska Native, Asian or Other Pacific Islander, or mixed races). We viewed race and ethnicity as societal constructs, not deterministic biological causes of disease risk, and included them as proxy measures of structural racism that may be associated with access to residential green space and resources that promote healthy weight.^29,30^ We calculated maternal prepregnancy BMI using self-reported prepregnancy weight and height. Mothers reported their highest educational attainment and that of their partner in questionnaires in early pregnancy (college degree: yes or no) and their household income and marital status in the mid-childhood and early adolescence visit. Household income was categorized as more than $70 000 per year or $70 000 per year or less based on the distribution of income categories,^31^ and marital status was categorized as married or cohabiting (yes or no). Area-level covariates at mid-childhood and early adolescence included neighborhood socioeconomic status (SES) based on 2010 US Census tract–level median annual household income (continuously; US dollars) and 2010 US Census tract–level population density (continuously; persons per kilometer squared). We chose these covariates based on a directed acyclic graph (eFigure 2 in Supplement 1),^32^ supported by prior literature.^8,33^

### Statistical Analysis

Data were analyzed from January 1 to June 30, 2024. Statistical tests were 2-sided. We applied linear regression models to quantify prospective associations of per SD increment in street-view (500-m street-view metrics) and satellite-based (270-m NDVI) green space exposures with all 4 adiposity measures (ie, BMI and waist circumference *z* scores and total and trunk FMI). We fitted separate models for each exposure period: (1) green space exposure in mid-childhood and early adolescence adiposity and (2) green space exposure in early adolescence and late adolescence adiposity. Given the strong correlation between the NDVI and street-view trees (*r* = 0.84) and grass (*r* = 0.63), we examined NDVI and street-view measures using separate models (eFigure 3 in Supplement 1). In models for street-view metrics, we adjusted all 3 metrics (percentage of trees, grass, and other green space) mutually. This approach helps us understand the specific mechanisms by which each component may be associated with adiposity outcomes. Finally, we fitted generalized additive models to examine potential nonlinear associations (eFigure 4 in Supplement 1). However, penalized splines showed no deviations from linearity (*P* > .10); therefore, we present results from the linear models.

Our fully adjusted models included child’s age at baseline, sex, race and ethnicity, mother’s educational level, partner’s educational level, mother’s prepregnancy BMI, household income, neighborhood SES, and population density. We used multiple imputation by chained equations (“mice” package in R, version 4.3.0 [R Project for Statistical Computing]) to impute missing covariate data with 20 iterations under the missing-at-random assumption.^34^ We did not, however, impute missing exposure or outcome data, as they are highly likely to not be missing at random. The missing rates for covariates were mostly less than 5%, except for the partner’s educational level (10%). We then performed analyses across 20 imputed datasets and reported the pooled estimates. The results were broadly comparable with those obtained using the observed values; the former are presented herein.

#### Effect Modification

We examined effect modification by child’s sex, race and ethnicity (Hispanic, non-Hispanic Black, non-Hispanic White, or other), Census tract–level population density (tertiles of persons per kilometer squared; low, <518; medium, 518-912; high, >912), and Census tract SES (tertiles of median household income; low, <$53 500; medium, $53 500-72 100; high, >$72 100) based on previous studies.^20,35,36^ We fit models that included a multiplicative interaction term between each green space measure and the effect modifier, and we reported stratum-specific coefficients and 95% CI if significant (*P* < .05 for interaction).

#### Sensitivity Analyses

First, we tested the robustness of the model using different buffer sizes for street-view and satellite-based measures to assess green space exposure and by restricting the sample to participants who remained at the same address between visits (nonmovers). Second, we used mean estimates from mid-childhood to early adolescence for street-view and satellite-based measures to estimate the cumulative association with late adolescence adiposity. All statistical analyses were performed using R, version 4.3.0 (R Project for Statistical Computing).^37^

## Results

### Sample Characteristics

Of 1279 children, 843 (65.9%) participated in the mid-childhood visit (mean [SD] age, 7.9 [0.8] years) and were included in the prospective analysis between mid-childhood green space exposure and early adolescence adiposity measures. The sample included 423 girls (50.2%) and 420 boys (49.8%); 87 children (10.3%) were Hispanic, 130 children (15.4%) were non-Hispanic Black, 538 children (63.8%) were non-Hispanic White, and 88 children (10.4%) were other races and ethnicities; and 603 children (75.2%) had household income of more than $70 000 (Table). The demographic characteristics were largely similar for 599 of 1177 children (50.9%) who participated in the early adolescence visit and were included in the prospective analysis between early adolescence green space exposure and late adolescence adiposity measures (eTable 1 in Supplement 1). Children with higher exposure to NDVI greenness at mid-childhood were more likely to be non-Hispanic White; have mothers who were more likely to be married, have higher educational attainment, have household income of more than $70 000/year, and have a lower prepregnancy BMI; and reside in neighborhoods with higher Census tract–level median income and lower population density (Table). The analytic sample in mid-childhood (843 of 1279 [65.9%]) had slightly higher household incomes and resided in neighborhoods with slightly higher population densities than the excluded samples (eTable 2 in Supplement 1). Furthermore, the early adolescence analytic sample (599 of 1177 [50.9%]) were more likely to be female; Hispanic, non-Hispanic Black, or other race and ethnicity; and reside in areas with a higher population density than the excluded samples (eTable 3 in Supplement 1).

**Table.** Baseline Characteristics (at Mid-Childhood Visit) of Study Participants in Project Viva by Quartiles of Satellite-Based Normalized Difference Vegetation Indices

Variable	Overall (N = 843)	Quartile 1 (n = 217)	Quartile 2 (n = 198)	Quartile 3 (n = 208)	Quartile 4 (n = 220)
Child					
Age at mid-childhood, mean (SD), y	7.9 (0.8)	8.0 (0.9)	7.9 (0.8)	7.8 (0.7)	7.7 (0.7)
Race and ethnicity, No. (%)					
Hispanic	87 (10.3)	40 (18.4)	25 (12.6)	10 (4.8)	12 (5.5)
Non-Hispanic Black	130 (15.4)	69 (31.8)	34 (17.2)	18 (8.7)	9 (4.1)
Non-Hispanic White	538 (63.8)	86 (39.6)	115 (58.1)	154 (74.0)	183 (83.2)
Other^a^	88 (10.4)	22 (10.1)	24 (12.1)	26 (12.5)	16 (7.3)
Sex, No. (%)					
Female	423 (50.2)	105 (48.4)	105 (53.0)	105 (50.5)	108 (49.1)
Male	420 (49.8)	112 (51.6)	93 (47.0)	103 (49.5)	112 (50.9)
Mother or family, No. (%)					
Completed college (mother)	586 (69.5)	108 (50.0)	131 (66.8)	162 (77.9)	185 (84.1)
Completed college (father)	521 (61.8)	85 (49.1)	110 (61.8)	149 (75.6)	177 (82.7)
Married or living together	714 (84.7)	154 (73.3)	167 (88.4)	190 (93.1)	203 (96.2)
Prepregnancy BMI, mean (SD)	24.9 (5.2)	25.8 (6.2)	25.2 (5.5)	24.3 (4.0)	24.1 (4.7)
Father’s BMI at enrollment, mean (SD)	26.5 (4.0)	26.6 (4.6)	26.4 (3.7)	26.5 (3.6)	26.6 (4.0)
Annual household income >$70 000, No. (%)	603 (71.4)	103 (50.0)	137 (74.9)	176 (87.1)	187 (88.6)
Neighborhood characteristics, mean (SD)					
Census tract median household income, $	86 482.4 (33 217.3)	62 621.1 (27 179.7)	77 950.6 (27 628.7)	96 531.8 (27 157.4)	108 195.7 (30 501.6)
Population density, persons/km^2^	2796.4 (3824.0)	6786.2 (5208.2)	2697.6 (2287.7)	1146.4 (959.2)	509.9 (520.5)
Green space measures at mid-childhood visit, mean (SD)					
% Trees (trees and palm trees)	28.2 (10.0)	17.7 (5.1)	24.5 (5.5)	30.8 (5.9)	39.2 (7.1)
% Grass	5.2 (3.5)	2.1 (1.9)	4.5 (3.0)	6.9 (3.1)	7.3 (2.9)
% Other green space (plants, fields, and flowers)	1.8 (0.8)	1.8 (0.8)	1.9 (0.7)	1.8 (0.7)	1.9 (0.9)
NDVI (range, 0-1)	0.6 (0.1)	0.4 (0.1)	0.6 (0.0)	0.7 (0.0)	0.8 (0.0)
Green space measures at early adolescence visit, mean (SD)					
% Trees (trees and palm trees)	28.7 (10.2)	19.3 (6.9)	25.1 (6.2)	31.2 (6.9)	38.9 (7.9)
% Grass	5.9 (3.4)	2.9 (2.4)	5.6 (3.0)	7.6 (2.8)	7.5 (2.9)
% Other green space (plants, fields, and flowers)	2.3 (0.9)	2.3 (0.9)	2.4 (0.7)	2.2 (0.9)	2.1 (1.0)
NDVI (range, 0-1)	0.6 (0.1)	0.5 (0.1)	0.6 (0.1)	0.7 (0.1)	0.8 (0.1)
Adiposity measures at early adolescence visit, mean (SD)					
BMI	20.8 (4.4)	22.0 (5.4)	20.7 (4.5)	20.1 (3.6)	20.2 (3.7)
Waist circumference, cm	72.6 (11.6)	74.6 (13.3)	72.6 (12.0)	71.4 (10.3)	71.7 (10.2)
Total FMI	6.3 (3.0)	6.8 (3.6)	6.2 (3.0)	6.0 (2.6)	6.0 (2.6)
Trunk FMI	2.4 (1.4)	2.6 (1.7)	2.4 (1.5)	2.3 (1.2)	2.3 (1.2)
Adiposity measures at late adolescence visit, mean (SD)					
BMI	23.9 (5.2)	25.6 (6.9)	23.3 (4.4)	23.6 (4.3)	22.9 (4.0)
Waist circumference, cm	81.5 (12.9)	84.7 (15.9)	80.3 (11.9)	81.0 (11.4)	80.0 (11.5)
Total FMI	7.1 (3.6)	7.9 (4.7)	7.0 (3.2)	6.9 (3.2)	6.4 (2.7)
Trunk FMI	2.9 (1.9)	3.4 (2.4)	2.9 (1.7)	2.9 (1.6)	2.6 (1.4)

Abbreviations: BMI, body mass index (calculated as weight in kilograms divided by height in meters squared); FMI, fat mass index (calculated as fat mass in kilograms divided by height in meters squared); NDVI, normalized difference vegetation index.

^a^ American Indian or Alaska Native, Asian or Pacific Islander, or mixed races.

### NDVI Green Space and Adiposity

**Figure 1.**
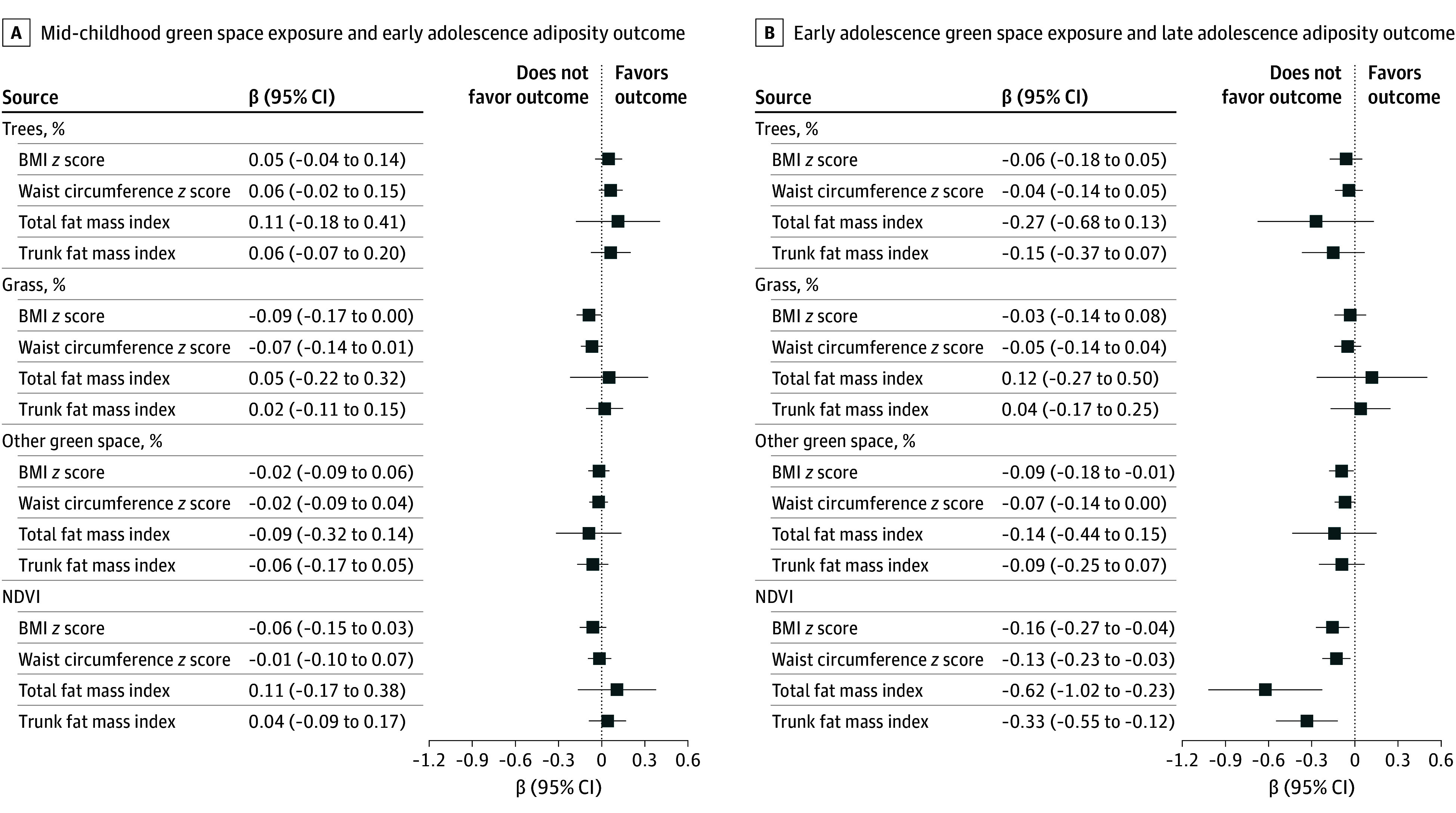
Estimated β Coefficients of 2 Prospective Associations of Street-View and Satellite-Based Green Space Measures (Per SD Increment) With 4 Adiposity Measures Among Children From Project Viva A, Mid-childhood green space exposure and early adolescence adiposity outcome. B, Early adolescence green space exposure and late adolescence adiposity outcome. Percentages of trees, grass, and other green space are mutually adjusted. Additional covariates included age, sex, race and ethnicity, prepregnancy body mass index (BMI), maternal educational level, paternal educational level, marital status, household income, neighborhood median income, and population density. The normalized difference vegetation index (NDVI) was highly correlated with the street-view trees metric (*r* = 0.84); therefore, they were fitted as separate models.

In unadjusted analyses (eFigure 5 in Supplement 1), higher levels of NDVI greenness within 270 m (per 1-SD increase) in mid-childhood were associated with lower BMI and waist circumference *z* scores and total and trunk FMI in early adolescence. We also observed similar inverse associations of NDVI in early adolescence with late adolescence adiposity. In fully adjusted models, associations of NDVI in mid-childhood with early adolescence adiposity were in the same direction but attenuated to the null. However, higher levels of NDVI in early adolescence remained associated with lower *z* scores for BMI (β, −0.16 [95% CI, −0.27 to −0.04]) and waist circumference (β, −0.13 [95% CI, −0.23 to −0.03]), lower total FMI (β, −0.62 [95% CI, −1.02 to −0.23), and lower trunk FMI (β, −0.33 [95% CI, −0.55 to −0.12) in late adolescence (Figure 1). The results were consistent when we examined the NDVI within 1230-m and 90-m buffers (eFigure 6A and 6B in Supplement 1), applied cumulative NDVI exposures (eFigure 6C in Supplement 1), used datasets without multiple imputation (eFigure 6D in Supplement 1), and restricted the sample to participants who remained at the same address (eFigure 6E in Supplement 1).

### Street-View Green Space and Adiposity

In unadjusted analyses (eFigure 5 in Supplement 1), we observed inverse associations of street-view trees in mid-childhood with total and trunk FMI in early adolescence. We also observed that higher percentages of street-view trees in early adolescence were associated with lower total and trunk FMI in late adolescence, while higher percentages of street-view other green space (plants, flowers, and fields) in early adolescence were associated with lower BMI or waist circumference *z* scores in late adolescence. In fully adjusted models, higher levels of street-view other green space within 500 m in early adolescence remained associated with lower *z* scores for BMI (β, −0.09 [95% CI, −0.18 to −0.01]), but the observed inverse associations for street-view trees with FMI measures were attenuated to the null for both prospective associations. These results were largely consistent when we examined street-view metrics within the 1000-m and 100-m buffer (eFigure 6A and 6B in Supplement 1), applied cumulative street-view exposures (eFigure 6C in Supplement 1), used datasets without multiple imputation (eFigure 6D in Supplement 1), and restricted the sample to participants who remained at the same address (eFigure 6E in Supplement 1).

### Effect Modification Analyses

**Figure 2.**
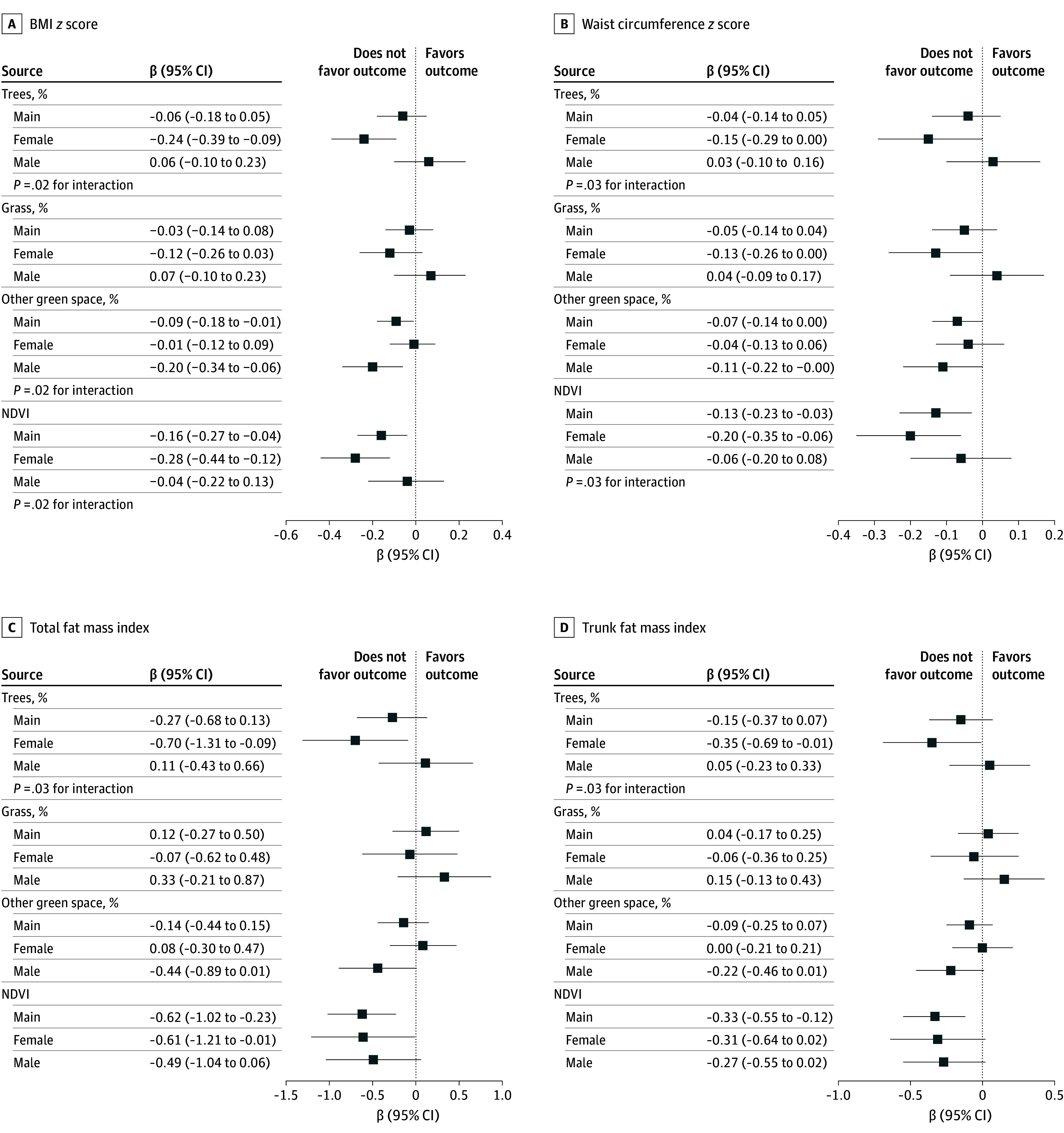
Effect Modification by Child’s Sex of the Association of Street-View and Satellite-Based Green Space Measures (Per SD Increment) at the Early Adolescence Visit With 4 Adiposity Measures in Late Adolescence in Children From Project Viva A, Body mass index (BMI) *z* score. B, Waist circumference *z* score. C, Total fat mass index. D, Trunk fat mass index. Percentages of trees, grass, and other green space are mutually adjusted. Additional covariates included age, sex, race and ethnicity, prepregnancy BMI, maternal educational level, paternal educational level, marital status, household income, neighborhood median income, and population density. The normalized difference vegetation index (NDVI) was highly correlated with the street-view trees metric (*r* = 0.84); therefore, they were fitted as separate models.

We found stronger inverse associations of street-view trees at the early adolescence visit with all adiposity measures in late adolescence among female participants for all 4 adiposity measures (Figure 2). For instance, each SD increase in percentages of street-view trees was associated with a −0.70 (95% CI, −1.31 to −0.09) lower total FMI among girls compared with a 0.11 (95% CI, −0.43 to 0.66) higher total FMI among boys (*P* = .03 for interaction). The association of higher NDVI with lower BMI and waist circumference *z* scores was also stronger in girls (Figure 2). The effect modification results by child’s sex for mid-childhood street-view trees and NDVI with early adolescence adiposity outcomes were largely similar (eFigure 7 in Supplement 1). Conversely, we found a stronger inverse association of street-view other green space at the early adolescence visit with BMI *z* scores in boys (β, −0.20 [95% CI, −0.34 to −0.06]; *P* = .02 for interaction) compared with girls (β, −0.01 [95% CI, −0.12 to 0.09]). We found no evidence of effect modification by race and ethnicity, neighborhood SES, or population density for associations of any of the satellite-based or street-view exposures in early adolescence with late adolescence adiposity outcomes (eFigure 8 in Supplement 1).

## Discussion

In this cohort study of US children, higher levels of satellite-based NDVI green space within 270 m of the participants’ homes in early adolescence were associated with lower BMI, waist circumference, and total body and trunk FMI in late adolescence. In addition, more street-view other green space (ie, flowers, plants, and fields) within 500 m of participants’ homes in early adolescence was associated with a lower BMI in late adolescence. Contrary to our initial hypothesis, the NDVI was more strongly and robustly associated with adiposity than street-view green space measures. These findings were consistent regardless of the size of the neighborhood buffer used, and the association was stronger among girls for the NDVI and street-view trees and among boys for street-view other green space.

Our findings align with those of several prior studies reporting inverse associations between the NDVI and adiposity measures in childhood and adolescence. In a study of 3831 children and adolescents aged 3 to 16 years in Indiana, Bell et al^21^ found that a higher NDVI was associated with lower BMI *z* scores 2 years later. Daniels et al^22^ reported that a higher NDVI was associated with lower BMI *z* scores over an 8-year period among 51 873 African American children aged 6 to 19 years. Our results also resemble those of a Dutch study of 1505 children aged 12 to 16 years,^38^ which found inverse associations between residential NDVI and waist circumference in both the early and late adolescence visits.

Our study provides additional evidence of the associations between specific green space components from street-view images and adiposity measures. We found an inverse association between street-view other green space (fields, flowers, and plants) in early adolescence and BMI in late adolescence, adjusting for street-view trees and grass, as well as inverse trends between street-view other green space and waist circumference and total and trunk FMI in the same period, although the latter were attenuated after adjusting for covariates. Components of street-view other green space may be a stronger indicator of well-maintained local communities, social cohesion, and neighborhood affluence.^39^ Visual inspection of sample street-view images with a high proportion of other green space in our study suggests that these places are often heavily landscaped along sidewalks or in parks. Therefore, we speculate that street-view other green space may indicate better aesthetics and perceived quality of green space, which have been associated with higher physical activity levels in children.^40^ In addition, we found that street-view trees and grass were not associated with adiposity measures at any time point, which was unexpected given the strong correlation between the NDVI and street-view trees and grass metrics and many studies suggesting that trees might be associated with reduced obesity risk by mitigating environmental hazards, such as air pollutants, and offering restorative stress reduction benefits.^2^ We speculate that street tree canopies may be less associated with adiposity than the additional proportions of trees and grass captured by the NDVI measure, such as those in backyards, home gardens, off-street parks and playgrounds, and sports fields,^41^ which past studies have associated with capacity building (eg, physical activity opportunities) that could prevent obesity in children.^40,42^

Despite attenuated associations from mid-childhood to early adolescence, we observed consistent associations of both the NDVI and street-view other green space in early adolescence with adiposity measures in late adolescence. This finding suggests that exposure during early adolescence may be a more sensitive time window than mid-childhood for the association of neighborhood green space with subsequent adiposity. Our findings align with those of an Australian study^20^ of 4423 children aged 6 to 13 years, which reported that the association of the NDVI with lower waist circumference did not emerge until children entered adolescence. It is possible that the benefits associated with green space for adiposity require an accumulation of exposure during childhood,^20^ as suggested by our sensitivity analysis, which identified associations of cumulative exposure over 2 time points with adiposity. Alternatively, green space exposure at a younger age may depend largely on parents and may take place in locations other than home (eg, community installations and school).^43,44^ In contrast, adolescents have more autonomy to use green spaces in their neighborhoods for recreational and social activities, on average, than childen,^43^ and green space has been associated with reduced obesity risk through pathways such as increased physical activity, better sleep, and reduced stress.^33^

Our stratified analyses revealed that the association of higher amounts of street-view trees in early adolescence with lower adiposity in late adolescence may be pronounced among girls. Klinker et al^44^ studied Danish children and adolescents aged 11 to 16 years and found that boys spent a larger proportion of their time engaging in physical activity outside their neighborhood where green space was less prevalent, such as in indoor sports facilities, compared with girls. In addition, we observed that other green spaces in early adolescence had a stronger association with BMI in late adolescence among boys. In the same study, Klinker et al^44^ reported that boys spent more time engaged in active commuting, such as cycling or walking. Therefore, the aesthetics and quality of green space along streets could play a larger role in encouraging boys to engage in utilitarian physical activity, which may help prevent obesity.

The magnitude of reductions in BMI *z* scores per SD increment in the NDVI and street-view other green space in our study ranged from −0.62 to −0.13, which is similar to the magnitude of reductions in BMI *z* scores reported in lifestyle intervention trials among children and adolescents with obesity that resulted in clinically meaningful improvements in cardiometabolic health biomarkers, such as blood pressure, lipid profile, insulin levels, and aerobic fitness.^45,46^ Although more replication in more diverse populations is needed, these findings signify that improving green space access through urban planning and public health policy may be a useful strategy that can be combined with individualized interventions to prevent obesity and cardiometabolic disease among adolescents.

### Strengths and Limitations

Our study has some strengths, including the use of objective high-quality measures of adiposity, specifically total and trunk FMI derived from dual-energy x-ray absorptiometry, which better inform whole-body and abdominal adiposity than BMI does. Second, to our knowledge, this study is the first to use both satellite-based and street-view data to examine the association between green space exposure and childhood obesity. Our approach benefits from advances in computers, resulting in street-level exposure metrics corresponding to ground-level perspectives. This complements the commonly used satellite-based NDVI measures and allows for the investigation of both overall green space and specific components of green space in association with childhood obesity.^47^ Third, our study provides greater insight into longer-term associations of green space with childhood obesity through prospective analyses.

Our study also has some limitations. Street-view images are snapshots of a location at a specific time and may not accurately represent the true green space over time with seasonal variability. Because street-view images are available only from 2007 onward, we could not assess street-view green space exposure before mid-childhood, which might also be associated with adiposity outcomes during childhood and adolescence. In addition, the study used images within 100 m, 500 m, and 1000 m of a participant’s address. It is possible that the images used may not be representative of where participants actually spend time, such as near their school.^48^ Both of these limitations can contribute to measurement error and may bias results.^49,50^ Our green space measures also did not account for participants’ perceived exposure to green space, such as aesthetics, design, and functionality (eg, the availability of playgrounds or equipment), which may be relevant to health behaviors and psychological factors associated with obesity.^51,52^ Furthermore, although we included several measures of SES in our analyses, our results may still be subject to residual confounding by other unmeasured social factors.^53^ Finally, Project Viva is predominantly composed of non-Hispanic White participants of higher SES, and most were still located in Massachusetts. Therefore, the results of our study may not be generalizable to other racial and ethnic groups and populations or other countries with different outdoor natural spaces.

## Conclusions

In this cohort study of US children, we observed prospective inverse associations of overall residential satellite-based green space and specific street-level green space components (flowers, plants, and fields) with adiposity measures among children. The results were sensitive to the growth periods of children, where we observed associations only between early and late adolescence, but not between mid-childhood and early adolescence, and may potentially differ by sex. Overall, the findings support the exploration of increasing residential green space levels and adding specific green space components as a promising urban planning and public health intervention strategy to combat the high prevalence of childhood obesity in the US.

## Notes

Supplement 1. **eTable 1.** A Comparison of Baseline Characteristics (Mid-Childhood vs Early Adolescence) of Study Participants in Project Viva **eTable 2.** A Comparison of Baseline Characteristics Study Participants Participated in Mid-Childhood Study in Project Viva (N = 1,279) With Those Excluded (N=436) and Included (N=843) in the Analytical Samples for the Prospective Analysis Between Mid-Childhood Greenspace Exposure and Early Adolescent Adiposity Outcomes **eTable 3.** A Comparison of Baseline Characteristics Study Participants Participated in Mid-Childhood Study in Project Viva (N = 1,177) With Those Excluded (N=578) and Included (N=599) in the Analytical Samples for the Prospective Analysis Between Early Adolescent Greenspace Exposure and Late Adolescent Adiposity Outcomes **eFigure 1.** Flowchart for Creation of Analytical Samples to Assess the Association of Mid-Childhood Greenspace Exposures With Early Adolescent Adiposity (N=843) and the Association of Early Adolescent Greenspace Exposure With Late Adolescent Adiposity (N=599) in Project Viva Participants **eFigure 2.** A Directed Acyclic Graph on Choosing Covariates and Confounders for the Study **eFigure 3.** Spearman Correlation Coefficients Among Street-View Metrics, NDVI, and Other Individual and Neighborhood Covariates Measured at Baseline (Mid-Childhood Visit) **eFigure 4.** Plots of Generalized Additive Model to Examine Potential Nonlinear Associations for Two Prospective Associations of Satellite-Based and Street-View Greenspace Exposures With Four Adiposity Measures **eFigure 5.** Estimated Beta Coefficients (95% CI) of Two Prospective Associations (Without Adjusting for Individual and Area-Level Covariates) of Street-View and Satellite-Based Greenspace Exposures (Per SD Increment) With Four Adiposity Measures in Children From Project Viva **eFigure 6.** Estimates of Associations of Street-View and Satellite-Based Greenspace Measures at the Early Adolescent Visit (Per SD Increment) With Four Adiposity Measures in Late Adolescence in Children From Project Viva Based on Full Model With (a) Larger Buffer Sizes, (b) Smaller Buffer Sizes, (c) Cumulative Exposure Across Mid-Childhood and Early Adolescence, (d) Using Data Without Imputation, (e) Only Participants Stayed in the Same Address **eFigure 7.** Effect Modification by Child’s Sex of the Association of Street-View and Satellite-Based Greenspace Measures (Per SD Increment) at the Mid-Childhood Visit and Four Adiposity Measures in Early Adolescence in Children From Project Viva **eFigure 8.** Effect Modification by Race and Ethnicity, Tertiles of Neighborhood Socioeconomic Status (NSES), and Tertiles of Population Density (PopD) of the Association of Street-View and Satellite-Based Greenspace Measures at the Early Adolescent Visit and Four Adiposity Measures in Late Adolescence in Children From Project Viva
eMethods.

eReferences.
**eFigure 9.** Example Images With High Values of Street-View Trees, Grass, and Other Greenspace Metrics Identified **eTable 4.** List of Classification Labels Segmented by PSPNet ADE20K Algorithm

Supplement 2.
Data Sharing Statement
