# Supplementary material for: Satellite-Based and Street-View Green Space and Adiposity in US Children
Source: JAMA Netw Open. 2024 Dec 5;7(12):e2449113. doi: 10.1001/jamanetworkopen.2024.49113 (PMC11621986; doi:10.1001/jamanetworkopen.2024.49113)
Supplement: Supplement 1. — eTable 1. A Comparison of Baseline Characteristics (Mid-Childhood vs Early Adolescence) of Study Participants in Project Viva eTable 2. A Comparison of Baseline Characteristics Study Participants Participated in Mid-Childhood Study in Project Viva (N = 1,279) With Those Excluded (N=436) and Included (N=843) in the Analytical Samples for the Prospective Analysis Between Mid-Childhood Greenspace Exposure and Early Adolescent Adiposity Outcomes eTable 3. A Comparison of Baseline Characteristics Study Participants Participated in Mid-Childhood Study in Project Viva (N = 1,177) With Those Excluded (N=578) and Included (N=599) in the Analytical Samples for the Prospective Analysis Between Early Adolescent Greenspace Exposure and Late Adolescent Adiposity Outcomes eFigure 1. Flowchart for Creation of Analytical Samples to Assess the Association of Mid-Childhood Greenspace Exposures With Early Adolescent Adiposity (N=843) and the Association of Early Adolescent Greenspace Exposure With Late Adolescent Adiposity (N=599) in Project Viva Participants eFigure 2. A Directed Acyclic Graph on Choosing Covariates and Confounders for the Study eFigure 3. Spearman Correlation Coefficients Among Street-View Metrics, NDVI, and Other Individual and Neighborhood Covariates Measured at Baseline (Mid-Childhood Visit) eFigure 4. Plots of Generalized Additive Model to Examine Potential Nonlinear Associations for Two Prospective Associations of Satellite-Based and Street-View Greenspace Exposures With Four Adiposity Measures eFigure 5. Estimated Beta Coefficients (95% CI) of Two Prospective Associations (Without Adjusting for Individual and Area-Level Covariates) of Street-View and Satellite-Based Greenspace Exposures (Per SD Increment) With Four Adiposity Measures in Children From Project Viva eFigure 6. Estimates of Associations of Street-View and Satellite-Based Greenspace Measures at the Early Adolescent Visit (Per SD Increment) With Four Adiposity Measures in Late Adolescence in Children [file jamanetwopen-e2449113-s001.pdf]

Supplementary Online Content

Yi L, Harnois-Leblanc S, Rifas-Shiman SL, et al. Satellite-based and street-view greenspace and adiposity in US children. *JAMA Netw Open.* 2024;7(12):e2449113.
doi:10.1001/jamanetworkopen.2024.49113

eTable 1. A Comparison of Baseline Characteristics (Mid-Childhood vs Early Adolescence) of Study Participants in Project Viva
eTable 2. A Comparison of Baseline Characteristics Study Participants Participated in Mid-Childhood Study in Project Viva (N = 1,279) With Those Excluded (N=436) and Included (N=843) in the Analytical Samples for the Prospective Analysis Between Mid-Childhood Greenspace Exposure and Early Adolescent Adiposity Outcomes
eTable 3. A Comparison of Baseline Characteristics Study Participants Participated in Mid-Childhood Study in Project Viva (N = 1,177) With Those Excluded (N=578) and Included (N=599) in the Analytical Samples for the Prospective Analysis Between Early Adolescent Greenspace Exposure and Late Adolescent Adiposity Outcomes
eFigure 1. Flowchart for Creation of Analytical Samples to Assess the Association of Mid-Childhood Greenspace Exposures With Early Adolescent Adiposity (N=843) and the Association of Early Adolescent Greenspace Exposure With Late Adolescent Adiposity (N=599) in Project Viva Participants
eFigure 2. A Directed Acyclic Graph on Choosing Covariates and Confounders for the Study
eFigure 3. Spearman Correlation Coefficients Among Street-View Metrics, NDVI, and Other Individual and Neighborhood Covariates Measured at Baseline (Mid-Childhood Visit)
eFigure 4. Plots of Generalized Additive Model to Examine Potential Nonlinear Associations for Two Prospective Associations of Satellite-Based and Street-View Greenspace Exposures With Four Adiposity Measures
eFigure 5. Estimated Beta Coefficients (95% CI) of Two Prospective Associations (Without Adjusting for Individual and Area-Level Covariates) of Street-View and Satellite-Based Greenspace Exposures (Per SD Increment) With Four Adiposity Measures in Children From Project Viva
eFigure 6. Estimates of Associations of Street-View and Satellite-Based Greenspace Measures at the Early Adolescent Visit (Per SD Increment) With Four Adiposity Measures in Late Adolescence in Children From Project Viva Based on Full Model With (a) Larger Buffer Sizes, (b) Smaller Buffer Sizes, (c) Cumulative Exposure Across Mid-Childhood and Early Adolescence, (d) Using Data Without Imputation, (e) Only Participants Stayed in the Same Address
eFigure 7. Effect Modification by Child's Sex of the Association of Street-View and Satellite-Based Greenspace Measures (Per SD Increment) at the Mid-Childhood Visit and Four Adiposity Measures in Early Adolescence in Children From Project Viva
eFigure 8. Effect Modification by Race and Ethnicity, Tertiles of Neighborhood Socioeconomic Status (NSES), and Tertiles of Population Density (PopD) of the Association of Street-View and Satellite-Based Greenspace Measures at the Early Adolescent Visit and Four Adiposity Measures in Late Adolescence in Children From Project Viva
eMethods.
eReferences.
eFigure 9. Example Images With High Values of Street-View Trees, Grass, and Other Greenspace Metrics Identified
eTable 4. List of Classification Labels Segmented by PSPNet ADE20K Algorithm

This supplementary material has been provided by the authors to give readers additional information about their work.

© 2024 Yi L et al. *JAMA Network Open.*

eTable 1. A Comparison of Baseline Characteristics (Mid-Childhood vs Early Adolescence) of Study Participants in Project Viva

Variable	Mid-childhood Visit (N = 843)	Early Adolescent Visit (N=599)
**Child**		
Age at mid-childhood	7.9 (0.8)	13.0 (0.7)
Race and ethnicity		
Non-Hispanic White	63.8%	63.3%
Hispanic	10.3%	10.7%
Non-Hispanic Black	15.4%	14.5%
Other races	10.4%	11.5%
Female	50.2%	51.8%
**Mother/Family**		
Completed college (mother)	69.8%	72.4%
Completed college (father)	68.4%	70.1%
Married/Living together	87.7%	83.5%
Pre-pregnancy BMI (kg/m²)	24.9 (5.2)	24.8 (5.3)
Father's BMI at enrollment (kg/m²)	26.5 (4.0)	26.4 (4.1)
Annual household income >$70,000	75.2%	78.6%
**Neighborhood Characteristics**		
Census tract median household income ($)	86,482.4 (33,217.3)	88,068.4 (33,089.7)
Population density (persons per km²)	2,796.4 (3,824.0)	2,567.6 (3,477.1)
**Greenspace Measures**		
% Trees (trees + palm trees)	28.2 (10.0)	28.6 (10.2)
% Grass	5.2 (3.5)	6.0 (3.4)
% Other green (plants + fields + flowers)	1.8 (0.8)	2.3 (0.9)
Normalized Difference Vegetation Indices (NDVI; 0~1)	0.6 (0.1)	0.6 (0.1)
**Adiposity Measures**		
BMI (kg/m²)	20.8 (4.4)	23.9 (5.2)
Waist circumference (cm)	72.6 (11.6)	81.5 (13.0)
Total fat mass index (kg/m²)	6.3 (3.0)	7.1 (3.6)
Trunk fat mass index (kg/m²)	2.4 (1.4)	3.0 (1.9)

Notes. BMI = body mass index.

© 2024 Yi L et al. *JAMA Network Open.*

eTable 2. A Comparison of Baseline Characteristics Study Participants Participated in Mid-Childhood Study in Project Viva (N = 1,279) With Those Excluded (N=436) and Included (N=843) in the Analytical Samples for the Prospective Analysis Between Mid-Childhood Greenspace Exposure and Early Adolescent Adiposity Outcomes

Variable	Overall<br>N = 1,279	Excluded sample<br>N = 436	Included sample<br>N = 843
**Child**			
Age at mid-childhood	8.0 (0.8)	8.2 (0.9)	7.9 (0.8)
Race and ethnicity			
Non-Hispanic White	64.5%	65.7%	63.8%
Hispanic	9.9%	9.2%	10.3%
Non-Hispanic Black	15.3%	15.2%	15.4%
Other races	10.3%	9.9%	10.4%
Female	49.6%	48.4%	50.2%
**Mother/Family**			
Completed college (mother)	69.3%	68.4%	69.8%
Completed college (father)	68.4%	68.6%	68.4%
Married/Living together	87.7%	87.5%	87.7%
Pre-pregnancy BMI (kg/m²)	24.7 (5.2)	24.3 (5.0)	24.9 (5.2)
Father's BMI at enrollment (kg/m²)	26.4 (4.0)	26.1 (3.9)	26.5 (4.0)
Annual household income >$70,000	74.2%	71.8%	75.2%
**Neighborhood Characteristics**			
Census tract median household income ($)	86,799.5 (34,643.5)	87,510.3 (37,682.3)	86,482.4 (33,217.3)
Population density (persons per km²)	2,613.7 (3,684.7)	2,204.0 (3,319.8)	2,796.4 (3,824.0)

Notes. BMI = body mass index.

© 2024 Yi L et al. *JAMA Network Open.*

eTable 3. A Comparison of Baseline Characteristics Study Participants Participated in Mid-Childhood Study in Project Viva (N = 1,177) With Those Excluded (N=578) and Included (N=599) in the Analytical Samples for the Prospective Analysis Between Early Adolescent Greenspace Exposure and Late Adolescent Adiposity Outcomes

Variable	Overall<br>N = 1,177	Excluded sample<br>N = 578	Included sample<br>N = 599
**Child**			
Age at mid-childhood	13.3 (1.0)	13.6 (1.1)	13.0 (0.7)
Race and ethnicity			
Non-Hispanic White	65.1%	67.0%	63.3%
Hispanic	9.9%	9.2%	10.7%
Non-Hispanic Black	14.7%	14.9%	14.5%
Other races	10.3%	9.0%	11.5%
Female	48.3%	44.8%	51.8%
**Mother/Family**			
Completed college (mother)	72.0%	71.5%	72.4%
Completed college (father)	69.8%	69.4%	70.1%
Married/Living together	83.3%	83.1%	83.5%
Pre-pregnancy BMI (kg/m²)	24.8 (5.3)	24.8 (5.3)	24.8 (5.3)
Father's BMI at enrollment (kg/m²)	26.4 (4.0)	26.5 (3.9)	26.4 (4.1)
Annual household income >$70,000	77.4%	76.1%	78.6%
**Neighborhood Characteristics**			
Census tract median household income ($)	89,130.0 (35,130.7)	90,269.7 (37,193.5)	88,068.4 (33,089.7)
Population density (persons per km²)	2,302.1 (3,185.5)	2,017.1 (2,814.8)	2,567.6 (3,477.1)

Notes. BMI = body mass index.

© 2024 Yi L et al. *JAMA Network Open.*

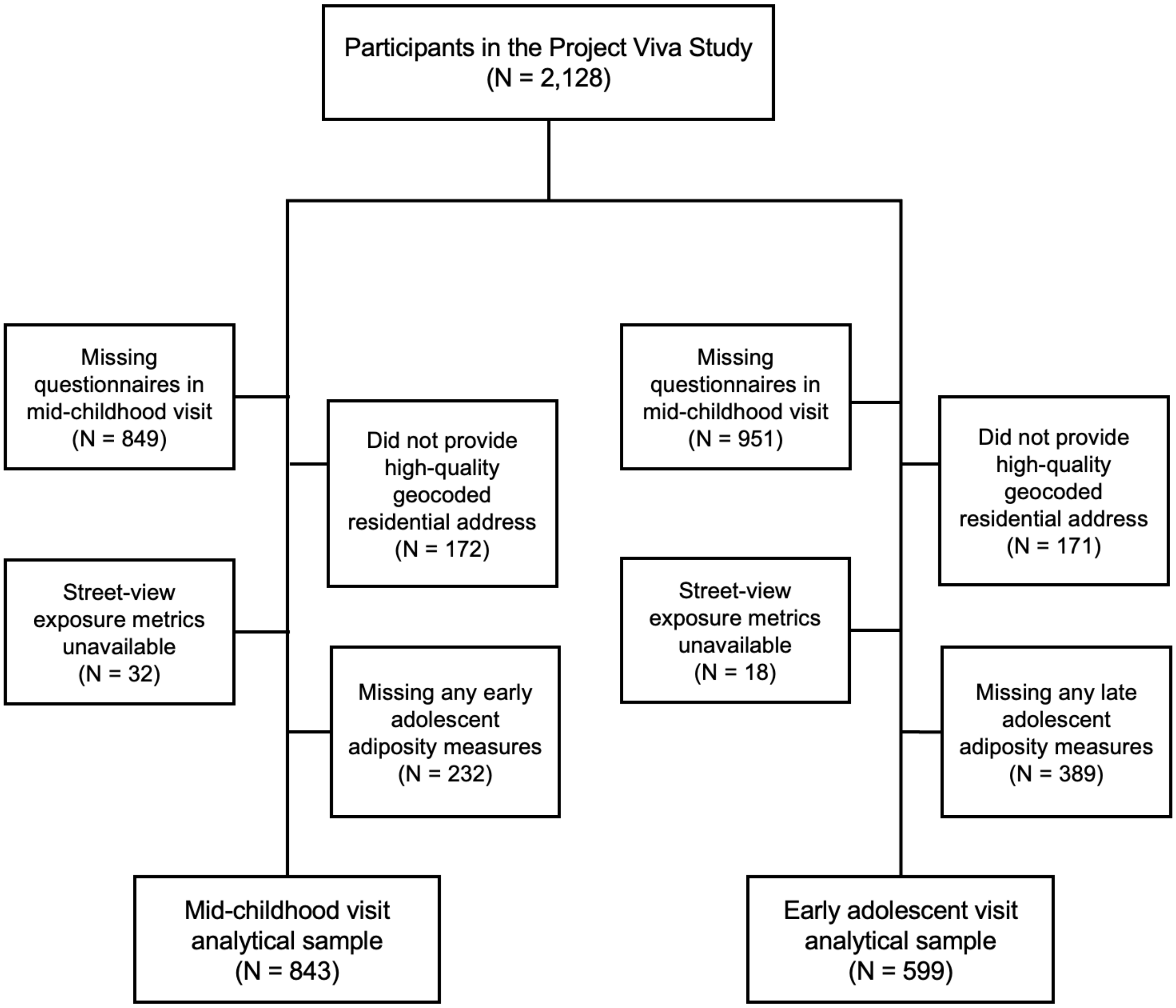

eFigure 1. Flowchart for Creation of Analytical Samples to Assess the Association of Mid-Childhood Greenspace Exposures With Early Adolescent Adiposity (N=843) and the Association of Early Adolescent Greenspace Exposure With Late Adolescent Adiposity (N=599) in Project Viva Participants

© 2024 Yi L et al. *JAMA Network Open.*

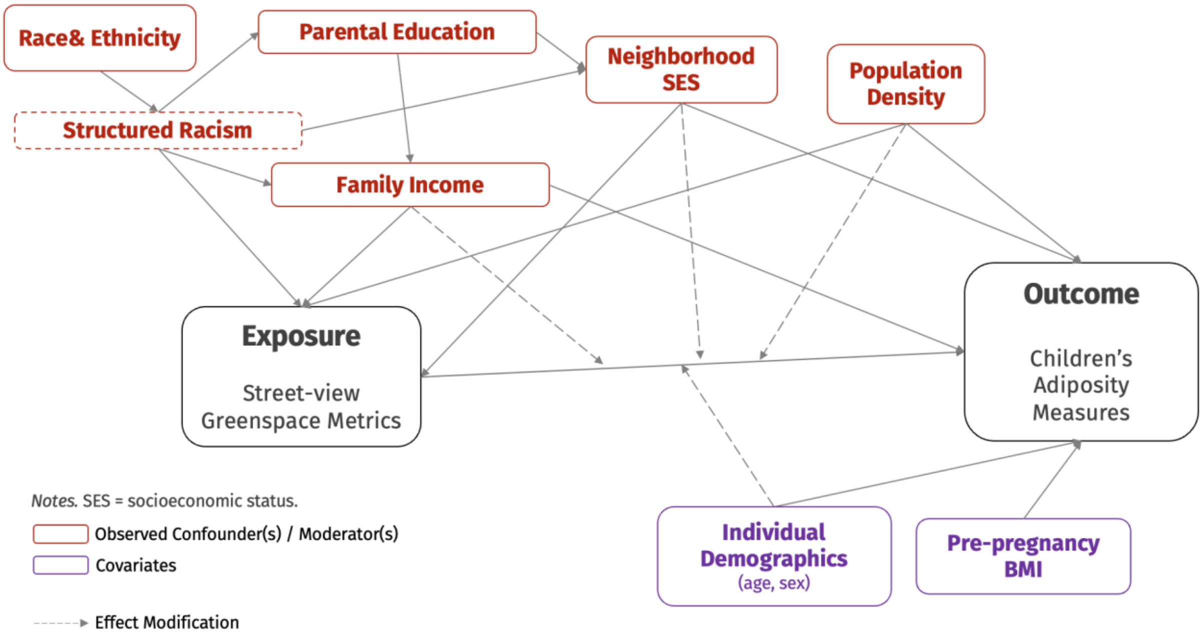

eFigure 2. A Directed Acyclic Graph on Choosing Covariates and Confounders for the Study

© 2024 Yi L et al. *JAMA Network Open.*

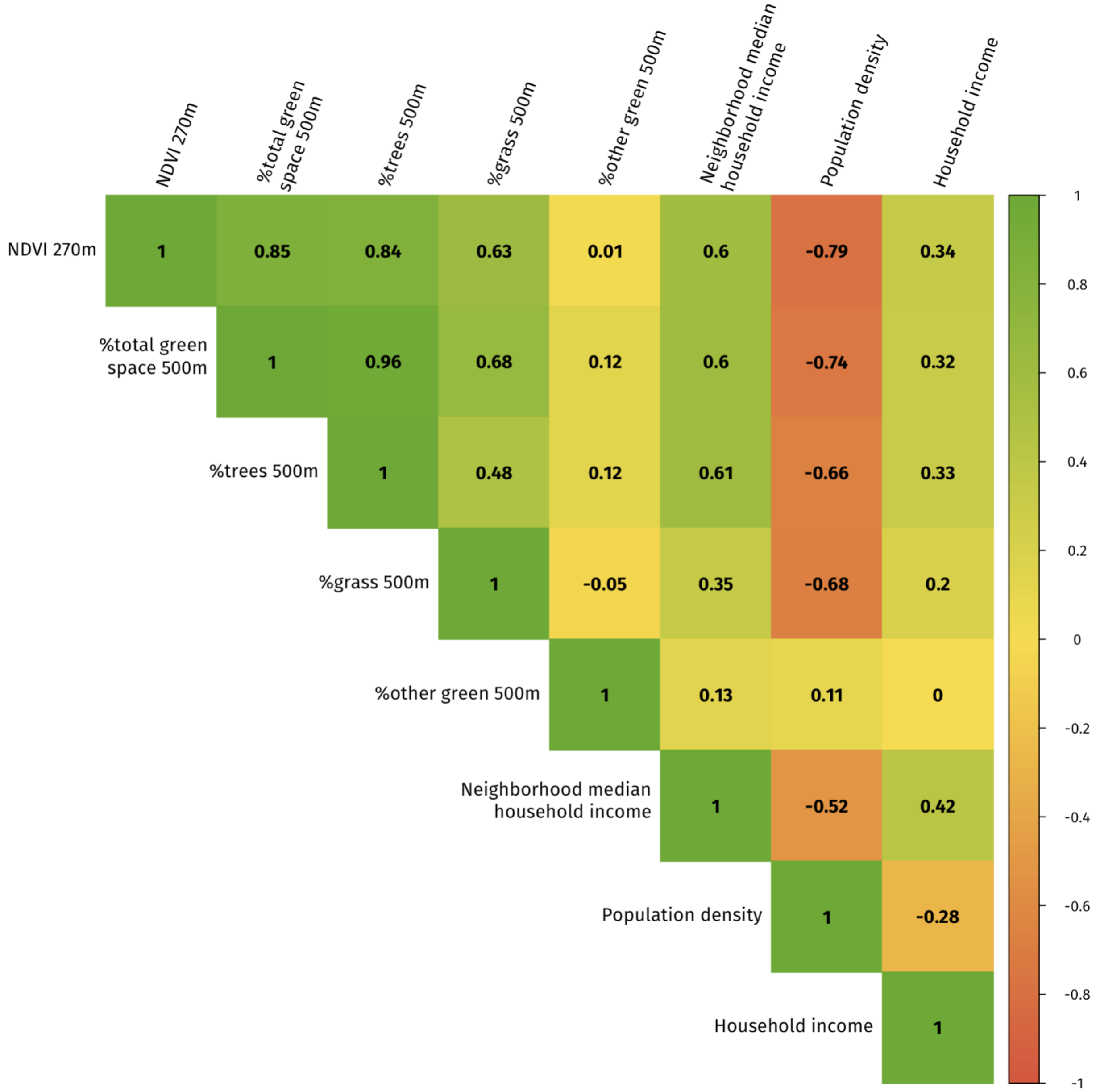

eFigure 3. Spearman Correlation Coefficients Among Street-View Metrics, NDVI, and Other Individual and Neighborhood Covariates Measured at Baseline (Mid-Childhood Visit)

NDVI = normalized difference vegetation index. "Household income" is a self-reported variable with ">$70,000/y" coded as 1 and less or equal coded as 0.

© 2024 Yi L et al. *JAMA Network Open.*

(a) Early adolescent Greenspace Exposure ~ Late Adolescent Adiposity Outcomes

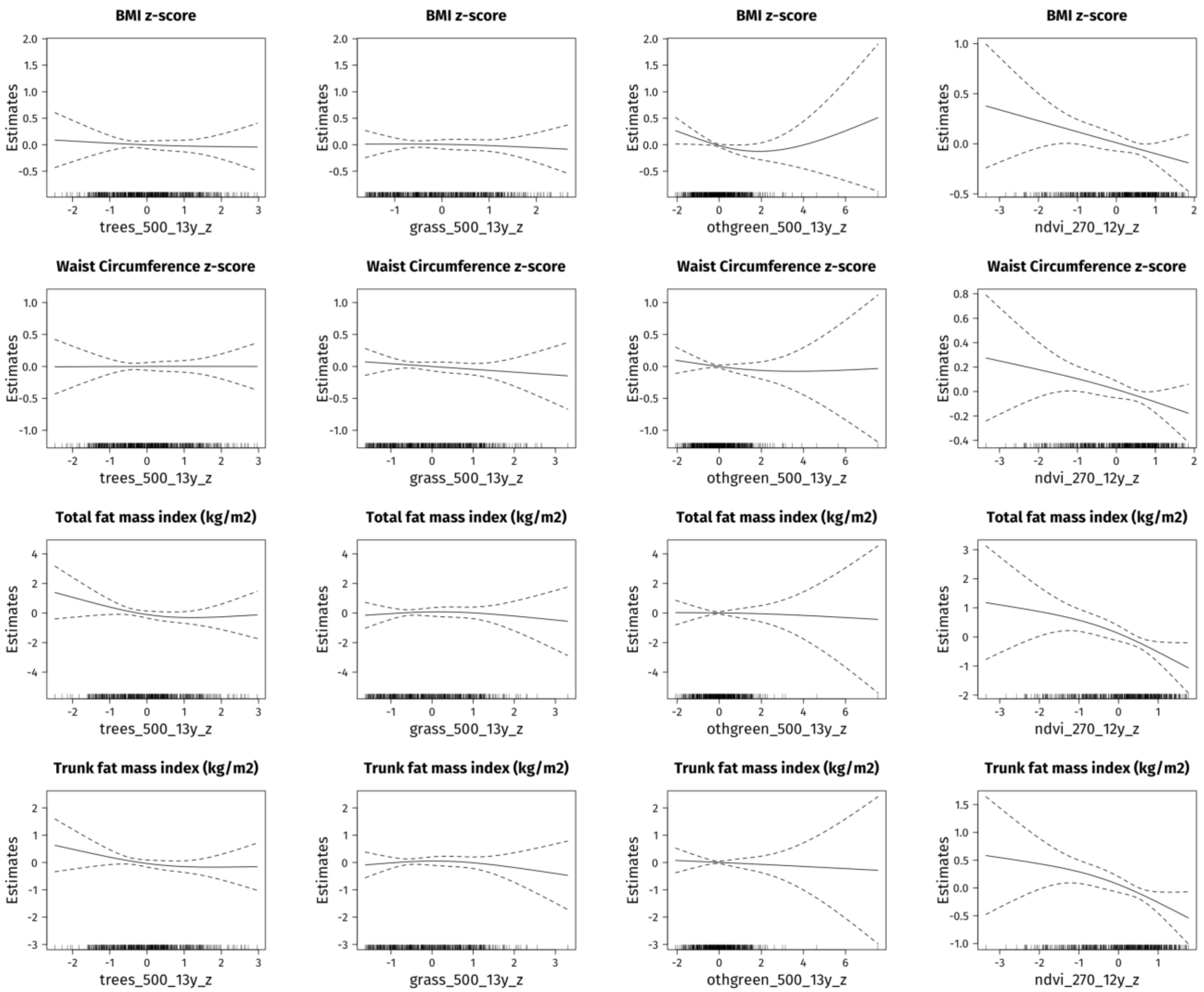

© 2024 Yi L et al. *JAMA Network Open.*

(b) Mid-childhood Greenspace Exposure ~ Early Adolescent Adiposity Outcomes

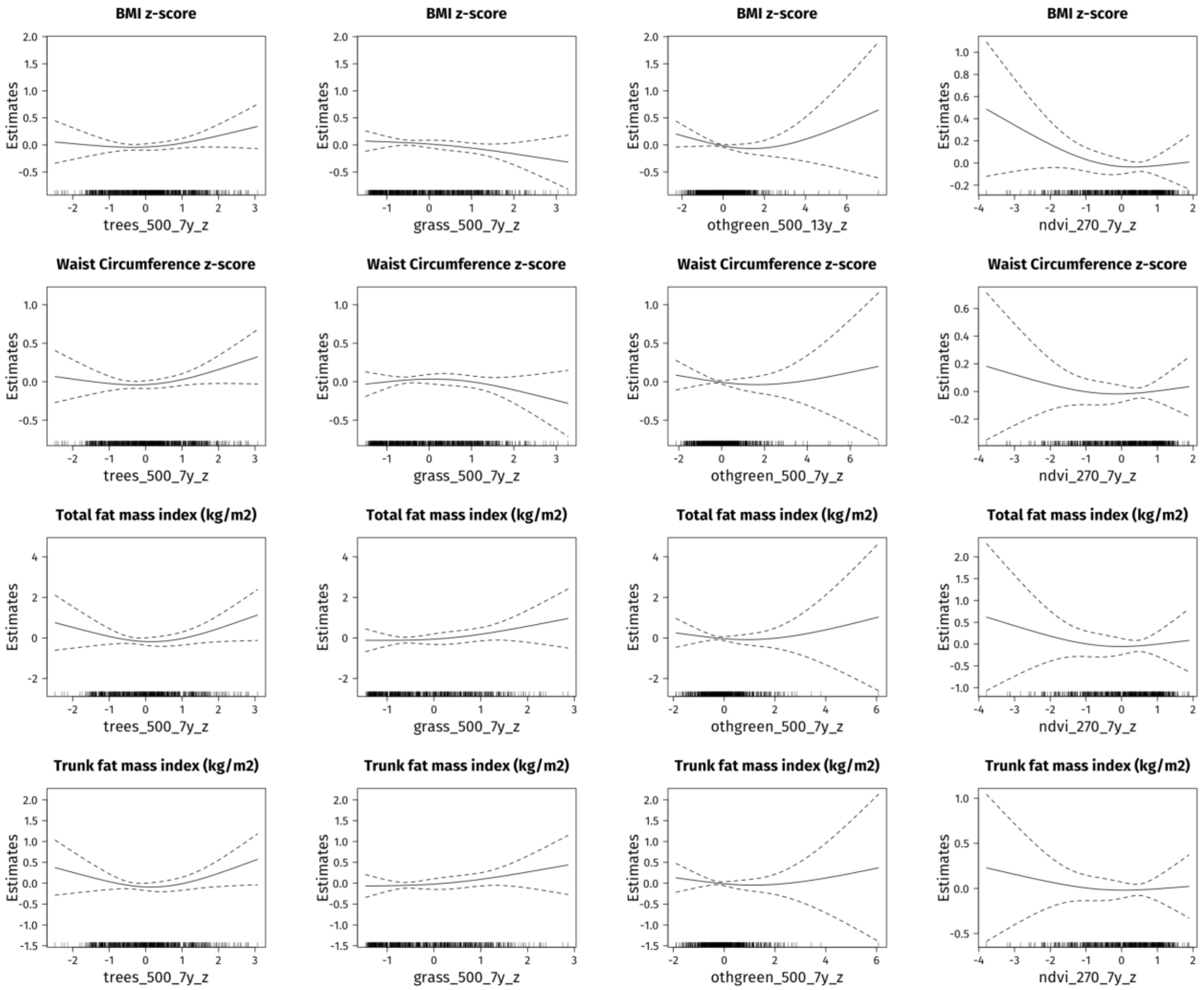

eFigure 4. Plots of Generalized Additive Model to Examine Potential Nonlinear Associations for Two Prospective Associations of Satellite-Based and Street-View Greenspace Exposures With Four Adiposity Measures

BMI = body mass index. NDVI = normalized difference vegetation index.

0

© 2024 Yi L et al. *JAMA Network Open.*

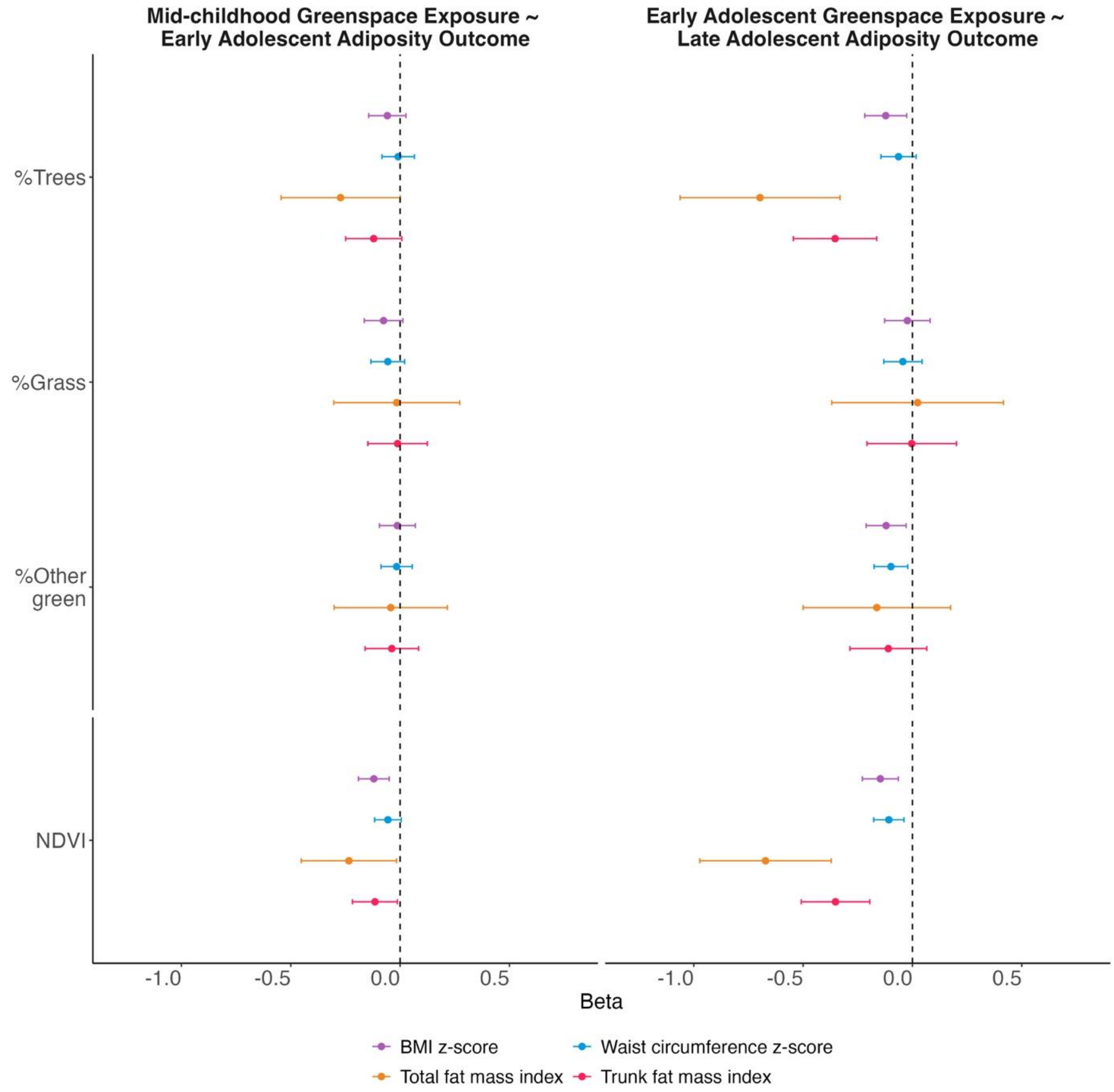

eFigure 5. Estimated Beta Coefficients (95% CI) of Two Prospective Associations (Without Adjusting for Individual and Area-Level Covariates) of Street-View and Satellite-Based Greenspace Exposures (Per SD Increment) With Four Adiposity Measures in Children From Project Viva

Notes.

1. %trees, %grass, and %other greenspace mutually adjusted
2. Models were unadjusted for other covariates
3. NDVI was highly correlated street-view trees metric (r = 0.84); therefore, they were fitted as separate models

BMI = body mass index. NDVI = normalized difference vegetation index.
* p<0.05; ** p<0.01; *** p<0.001

© 2024 Yi L et al. *JAMA Network Open.*

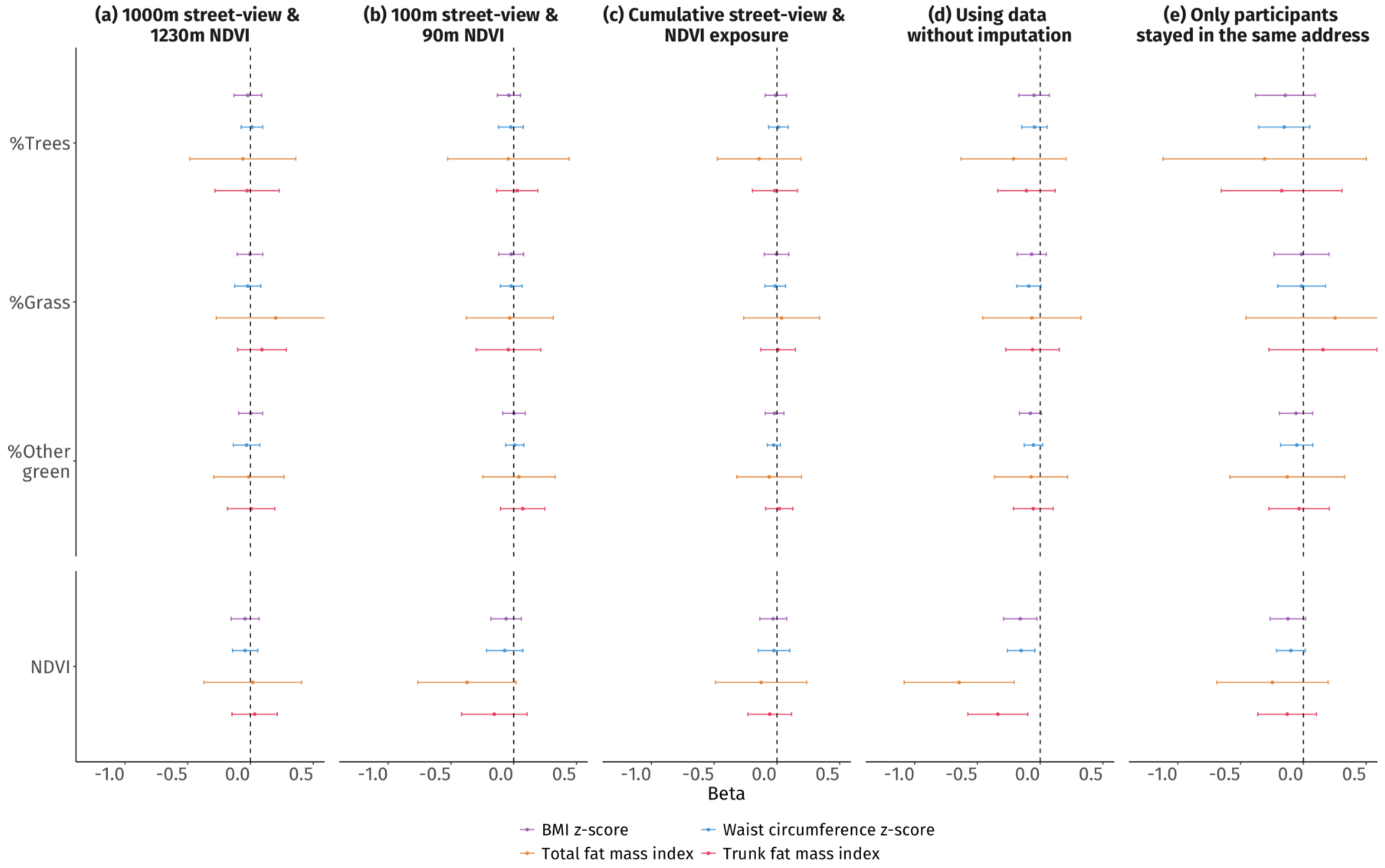

eFigure 6. Estimates of Associations of Street-View and Satellite-Based Greenspace Measures at the Early Adolescent Visit (Per SD Increment) With Four Adiposity Measures in Late Adolescence in Children From Project Viva Based on Full Model With (a) Larger Buffer Sizes, (b) Smaller

© 2024 Yi L et al. *JAMA Network Open.*

Buffer Sizes, (c) Cumulative Exposure Across Mid-Childhood and Early Adolescence, (d) Using Data Without Imputation, (e) Only Participants Stayed in the Same Address

Notes.

1. %trees, %grass, and %other greenspace mutually adjusted.
2. Additional covariates included age, sex, race and ethnicity, pre-pregnancy BMI, maternal education, paternal education, marital status, household income, neighborhood median income, and population density.
3. NDVI was highly correlated street-view trees metric (*r* = 0.84); therefore, they were fitted as separate models

BMI = body mass index. NDVI = normalized difference vegetation index.

2

© 2024 Yi L et al. *JAMA Network Open.*

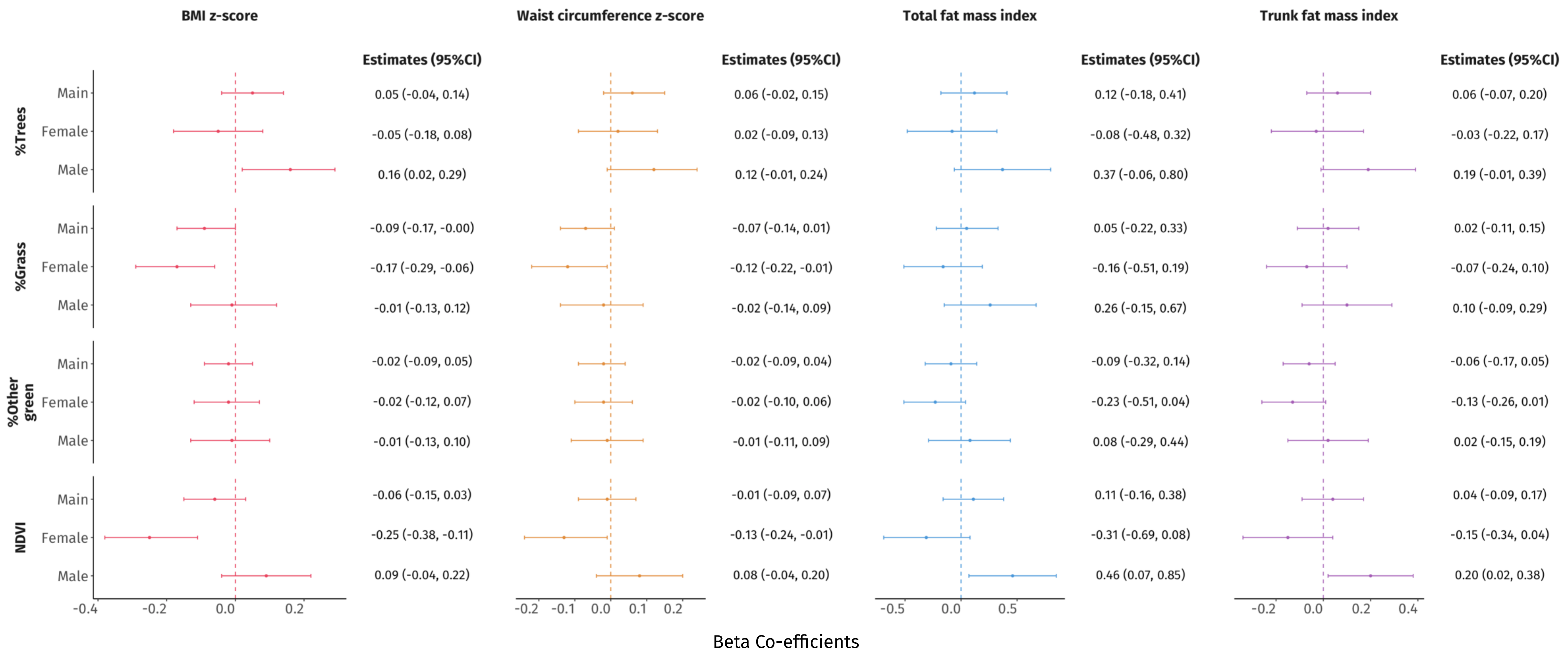

eFigure 7. Effect Modification by Child's Sex of the Association of Street-View and Satellite-Based Greenspace Measures (Per SD Increment) at the Mid-Childhood Visit and Four Adiposity Measures in Early Adolescence in Children From Project Viva

Notes.
1. %trees, %grass, and %other greenspace mutually adjusted.

© 2024 Yi L et al. *JAMA Network Open.*

2. Additional covariates included age, sex, race and ethnicity, pre-pregnancy BMI, maternal education, paternal education, marital status, household income, neighborhood median income, and population density.
3. NDVI was highly correlated street-view trees metric (*r* = 0.84); therefore, they were fitted as separate models.

BMI = body mass index. NDVI = normalized difference vegetation index.

© 2024 Yi L et al. *JAMA Network Open.*

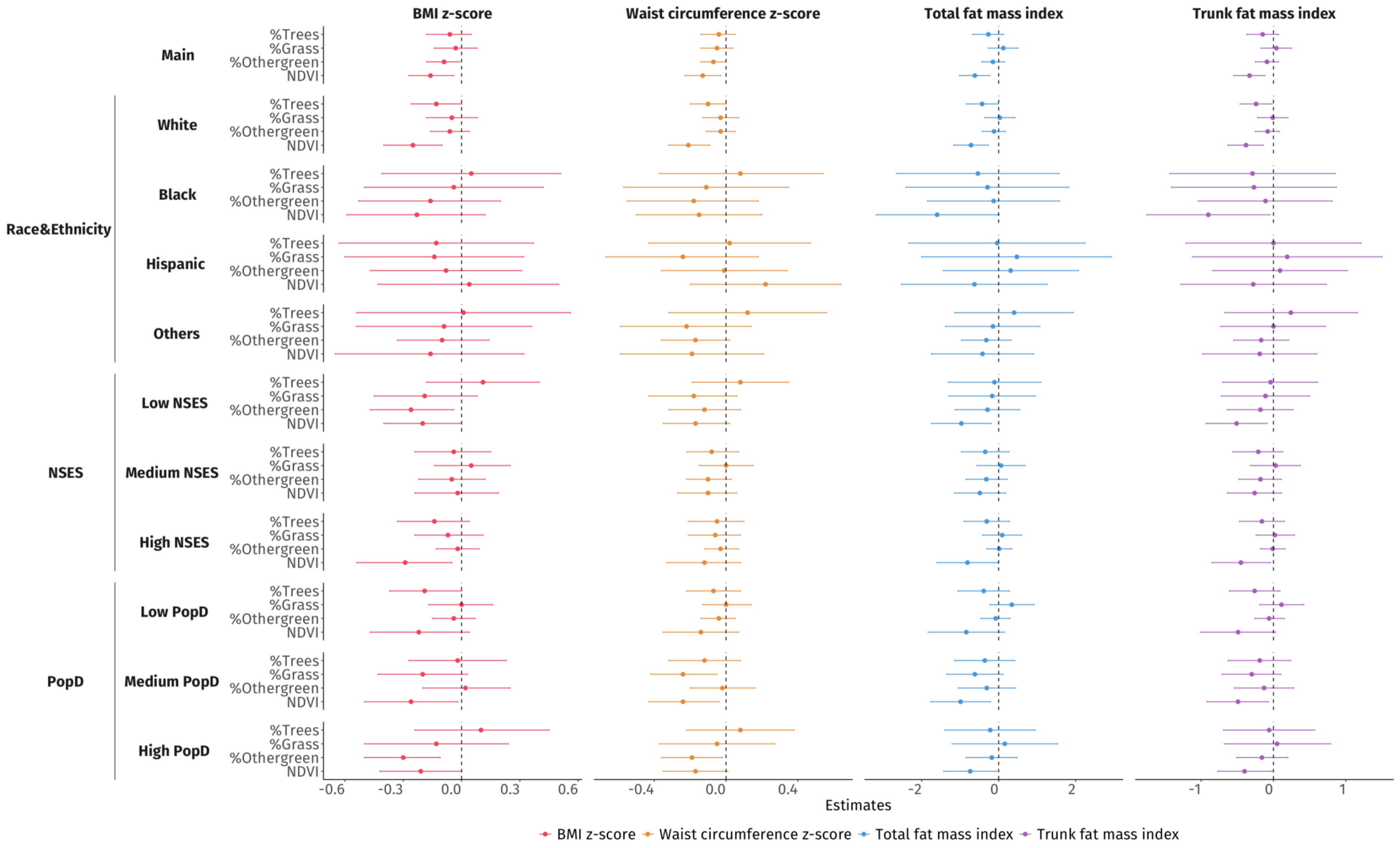

© 2024 Yi L et al. *JAMA Network Open.*

eFigure 8. Effect Modification by Race and Ethnicity, Tertiles of Neighborhood Socioeconomic Status (NSES), and Tertiles of Population Density (PopD) of the Association of Street-View and Satellite-Based Greenspace Measures at the Early Adolescent Visit and Four Adiposity Measures in Late Adolescence in Children From Project Viva

Notes.

1. %trees, %grass, and %other greenspace mutually adjusted.
2. Additional covariates included age, sex, race and ethnicity, pre-pregnancy BMI, maternal education, paternal education, marital status, household income, neighborhood median income, and population density, except for the stratifying variables.
3. NDVI was highly correlated street-view trees metric (r = 0.84); therefore, they were fitted as separate models.

BMI = body mass index. NDVI = normalized difference vegetation index

© 2024 Yi L et al. *JAMA Network Open.*

eMethods.

We created a 100-meter street network grid for every Census Bureau Statistical Area in the contiguous United States. Afterwards, we obtained all of the Google Street View (GSV) images from 2007 to 2020 within each street network grid. We chose to source the images from Google due to their consistency and thorough quality checks. To ensure a uniform standard of camera quality and positioning across all images, we exclusively used images captured by Google's street-view cars, which is essential for maintaining data integrity and facilitating accurate analysis. The majority of GSV images (approximately 78%) were collected from April to October. For each image location, we used four GSV images with different orientations to capture the horizontal street-level vision. To process all GSV images, we applied the pyramid scene parsing network (PSPNet), a deep learning approach pre-trained on the ADE20K dataset (Zhao et al., 2017). The ADE20K dataset comprises 150 pre-defined classes of objects and parts of objects (listed in the table below) and is described in detail elsewhere (Zhou et al., 2017; Bolei et al., 2017). PSPNet utilizes a convolutional neural network (Long et al., 2015; Hu et al., 2015; Nogueira et al., 2017) and incorporates both local (i.e., nearby pixels) and global (i.e., full image) contextual cues to produce more reliable pixel-level predictions. PSPNet has a strong model performance, achieving an overall pixel accuracy of 80.88% (Zhao et al., 2017).

Using PSPNet, we precisely segmented six greenspace features at the pixel level: trees, palm trees, grass, plants, fields, and flowers. We calculated the percentage coverage scores for each feature (e.g., the percentage of trees in an image). By averaging the percent coverage scores of images at a 100 m resolution raster for the contiguous US, we obtained an average value for each year. If a cell within a CBSA lacked a value in a specific year, we used the closest value in time if available, which was approximately 45% from the corresponding follow-up year ± 1 year and 72% from the corresponding follow-up year ± 3 years. In this study, we focused on three specific features: trees (including palm trees), other green features (including plants, fields, and flowers), and grass.

eReferences.

- Zhao H, Shi J, Qi X, Wang X, Jia J. Pyramid Scene Parsing Network. Published online 2017:2881-2890. Accessed September 28, 2023. https://github.com/hszhao/PSPNet.
- Zhou B, Zhao H, Puig X, Fidler S, Barriuso A, Torralba A. Scene Parsing Through ADE20K Dataset. Published online 2017:633-641. Accessed October 16, 2023. http://groups.csail.mit.edu/vision/datasets/ADE20K/.
- Bolei Z, Hang Z, Puig X, Fidler S, Barriuso A, Torralba A. ADE20K Dataset. Computer Vision and Pattern Recognition (CVPR). Published 2017. Accessed October 16, 2023. https://groups.csail.mit.edu/vision/datasets/ADE20K/.
- J. Long, E. Shelhamer, T. Darrell. Fully convolutional networks for semantic segmentation. Published Online (2015), pp. 3431-3440
- F. Hu, G.-S. Xia, J. Hu, *et al.*Transferring deep convolutional neural networks for the scene classification of high-resolution remote sensing imagery. Remote Sens., 7 (11) (2015), pp. 14680-14707, 10.3390/RS71114680
- K. Nogueira, O.A.B. Penatti, J.A. dos Santos. Towards better exploiting convolutional neural networks for remote sensing scene classification. Pattern Recognit., 61 (2017), pp. 539-556, 10.1016/J.PATCOG.2016.07.001

© 2024 Yi L et al. *JAMA Network Open.*

eFigure 9. Example Images With High Values of Street-View Trees, Grass, and Other Greenspace Metrics Identified

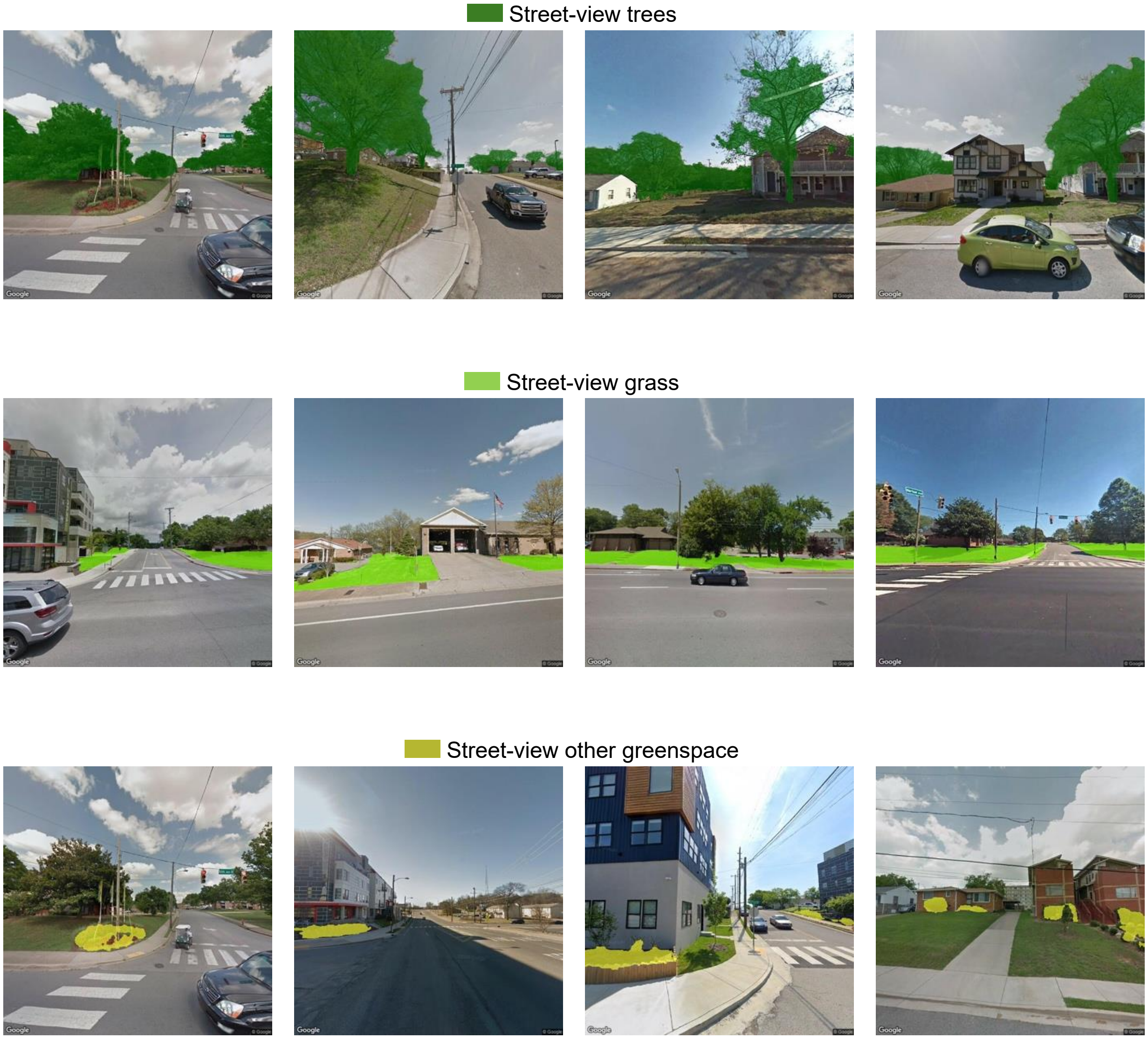

© 2024 Yi L et al. *JAMA Network Open.*

eTable 4. List of Classification Labels Segmented by PSPNet ADE20K Algorithm

ID	Class	ID	Class	ID	Class
**1**	wall	**51**	Refrigerator; icebox	**101**	poster; posting; placard; notice; bill; card
**2**	building; edifice	**52**	grandstand; covered; stand	**102**	stage
**3**	sky	**53**	path	**103**	van
**4**	floor; flooring	**54**	stairs; steps	**104**	ship
**5**	tree	**55**	runway	**105**	fountain
**6**	ceiling	**56**	case; display; case; showcase; vitrine	**106**	conveyer; belt; conveyor; belt; conveyer; conveyor; transporter
**7**	road; route	**57**	pool; table; billiard; table; snooker; table	**107**	canopy
**8**	bed	**58**	pillow	**108**	washer; automatic; washer; washing; machine
**9**	windowpane; window	**59**	screen; door; screen	**109**	plaything; toy
**10**	Grass	**60**	stairway; staircase	**110**	swimming; pool; swimming; bath; natatorium
**11**	cabinet	**61**	river	**111**	stool
**12**	sidewalk; pavement	**62**	bridge; span	**112**	barrel; cask
**13**	person; individual; someone; somebody; mortal; soul	**63**	bookcase	**113**	basket; handbasket
**14**	earth; ground	**64**	blind; screen	**114**	waterfall; falls
**15**	door; double; door	**65**	coffee; table; cocktail; table	**115**	tent; collapsible; shelter
**16**	table	**66**	toilet; can; commode; crapper; pot; potty; stool; throne	**116**	bag
**17**	mountain; mount	**67**	flower	**117**	minibike; motorbike
**18**	plant; flora; plant; life	**68**	book	**118**	cradle
**19**	curtain; drape; drapery; mantle; pall	**69**	hill	**119**	oven
**20**	chair	**70**	bench	**120**	ball
**21**	car; auto; automobile; machine; motorcar	**71**	countertop	**121**	food; solid; food
**22**	water	**72**	stove; kitchen; stove; range; kitchen; range; cooking; stove	**122**	step; stair
**23**	painting; picture	**73**	palm; palm; tree	**123**	tank; storage; tank
**24**	sofa; couch; lounge	**74**	kitchen; island	**124**	trade; name; brand; name; brand; marque
**25**	shelf	**75**	computer; computing; machine; computing; device; data; processor; electronic; computer; information; processing; system	**125**	microwave; microwave; oven
**26**	house	**76**	swivel; chair	**126**	pot; flowerpot
**27**	sea	**77**	boat	**127**	animal; animate; being; beast; brute; creature; fauna
**28**	mirror	**78**	bar	**128**	bicycle; bike; wheel; cycle
**29**	rug; carpet; carpeting	**79**	arcade; machine	**129**	lake
**30**	field	**80**	hovel; hut; hutch; shack; shanty	**130**	dishwasher; dish; washer; dishwashing; machine

© 2024 Yi L et al. *JAMA Network Open.*

**31**	armchair	**81**	bus; autobus; coach; charabanc; double-decker; jitney; motorbus; motorcoach; omnibus; passenger; vehicle	**131**	screen; silver; screen; projection; screen
**32**	seat	**82**	towel	**132**	blanket; cover
**33**	fence; fencing	**83**	light; light; source	**133**	sculpture
**34**	desk	**84**	truck; motortruck	**134**	hood; exhaust; hood
**35**	rock; stone	**85**	tower	**135**	sconce
**36**	wardrobe; closet; press	**86**	chandelier; pendant; pendent	**136**	vase
**37**	lamp	**87**	awning; sunshade; sunblind	**137**	traffic; light; traffic; signal; stoplight
**38**	bathtub; bathing; tub; bath; tub	**88**	streetlight; street; lamp	**138**	tray
**39**	railing; rail	**89**	booth; cubicle; stall; kiosk	**139**	ashcan; trash; can; garbage; can; wastebin; ash; bin; ash-bin; ashbin; dustbin; trash; barrel; trash; bin
**40**	cushion	**90**	television; television; receiver; television; set; tv; tv; set; idiot; box; boob; tube; telly; goggle; box	**140**	fan
**41**	base; pedestal; stand	**91**	airplane; aeroplane; plane	**141**	pier; wharf; wharfage; dock
**42**	box	**92**	dirt; track	**142**	crt; screen
**43**	column; pillar	**93**	apparel; wearing; apparel; dress; clothes	**143**	plate
**44**	signboard; sign	**94**	pole	**144**	monitor; monitoring; device
**45**	chest; of; drawers; chest; bureau; dresser	**95**	land; ground; soil	**145**	bulletin; board; notice; board
**46**	counter	**96**	bannister; banister; balustrade; balusters; handrail	**146**	shower
**47**	sand	**97**	escalator; moving; staircase; moving; stairway	**147**	radiator
**48**	sink	**98**	ottoman; pouf; pouffe; puff; hassock	**148**	glass; drinking; glass
**49**	skyscraper	**99**	bottle	**149**	clock
**50**	fireplace; hearth; open; fireplace	**100**	buffet; counter; sideboard	**150**	flag

© 2024 Yi L et al. *JAMA Network Open.*
