# Supplementary material for: Satellite-Based and Street-View Green Space and Adiposity in US Children
Source: JAMA Netw Open. 2024 Dec 5;7(12):e2449113. doi: 10.1001/jamanetworkopen.2024.49113 (PMC11621986; doi:10.1001/jamanetworkopen.2024.49113)
Supplement: Supplement 2. — Data Sharing Statement [file jamanetwopen-e2449113-s002.pdf]

Data Sharing Statement

Yi. Satellite-Based and Street-View Greenspace and Adiposity in US Children. *JAMA Netw Open*. Published December 05, 2024. doi:10.1001/jamanetworkopen.2024.49113

Data
Data available: No

Additional Information
Explanation for why data not available: Access to Project Viva data requires the proposal and approval of the specific research project and is not directly sharable. Details of obtaining access are available at projectviva.org
